# 

**Published:** 1908-06-20

**Authors:** 


### ST. BARTHOLOMEW'S HOSPITAL.

### RESIGNATION OF THE TREASURER AND ALMONERS.

The following announcement was published in the daily papers on Saturday, June 13:—Lord Ludlow (the treasurer) and the five almoners of St. Bartholomew's Hospital having resigned their posts in consequence of a unanimous recommendation made by them, and which they considered all important for the future welfare and good management of the hospital, not being adopted by the Court of Governors, a Court of Governors was held on the 11th inst., when the following resolution was unanimously adopted: "In accepting the resignation of the Right Hon. Lord Ludlow of the office of treasurer, to which he was elected on the 26th of January, 1905, this Court hereby records its deep sense of appreciation of the very valuable services he has rendered to the hospital. The important structural alterations and additions which have been carried out under his treasurership have added greatly to the efficiency of the hospital, and the advantages that have accrued to the institution as the result of the various reforms instituted by him in the management of the estates and in the internal administration of its affairs cannot be overestimated, and the Court tenders to Lord Ludlow its grateful acknowledgments." The almoners consented to postpone their resignation until July next in order that the affairs of the hospital may be carried on while the new officers are appointed.

### THE HOSPITAL SUNDAY FUND.

The Metropolitan Hospital Sunday Fund issued the following notice on June 10:—The Metropolitan Hospital Sunday Fund makes grants for the maintenance of patients and not for building. At the instance of the present Lord Mayor, invitations have been addressed this year to all the hospitals and institutions receiving awards to consult with the Distribution Committee before providing additional beds for patients, and this request has been well received. There are at present a very large number of beds empty for want of funds in existing hospitals, and unless the committees of hospitals see their way to maintain additional beds, it would be wrong to increase the present difficulty of providing funds. This year the difference between ordinary incomes and expenditure is about a quarter of a million, and this deficiency has only been met by expending legacies, an uncertain source of income that cannot be relied upon.

### THE GRADUATES' MEDICAL SCHOOLS.

DIARY FOR WEEK JUNE 22 TO 27.

### ROYAL COLLEGE OF PHYSICIANS, Pall Mall East.

Croonian Lectures.

**At 5 p.m.**
**June 23 and 25**, Dr. A. E. Garrod, **Inborn Errors in Metabolism.**

### THE HOSPITAL FOR SICK CHILDREN, Great Ormond Street, W.C.

**At 4 p.m.**
**June 25**, Mr. Steward, **Renal Tumours in Children.**

### THE POST GRADUATE COLLEGE, West London Hospital, Hammersmith, W.

**At 12 noon.**
**June 22**, Dr. Low, **Pathological Demonstration.**
**At 12.15 p.m.**
**June 24 and 26**, Dr. Pritchard, **Practical Medicine.**
**At 5 p.m.**
**June 22**, Mr. Bidwell, **Results after Gastroenterostomy.**
**June 23**, Dr. Low, **Ankylostomiasis.**
**June 24**, Dr. Beddard, **Medicine—IV.**
**June 25**, Mr. Edwards, **Clinical Lecture—II.**
**June 26**, Dr. Kenneth Scott, **Practical Ophthalmology.**

### NATIONAL HOSPITAL FOR THE PARALYSED AND EPILEPTIC, Queen Square, Bloomsbury.

**At 3.30 p.m.**
**June 23**, Dr. F. Buzzard, **Bulbar Paralysis.**
**June 26**, Dr. F. Buzzard, **Aphasia.**

### CHARING CROSS HOSPITAL, W.C.

**At 4 p.m.**
**June 25**, Mr. Daniel, **Surgical.**

### THE THROAT HOSPITAL, Golden Square, W.

**At 5.30 p.m.**
**June 22**, Dr. Bond, **Chronic Inflammatory Affections of the Oropharynx and Tonsils.**
**June 25**, Mr. Faulder, **Acute Inflammatory Affections of the Larynx.**

### NORTH-EAST LONDON POST-GRADUATE COLLEGE, Prince of Wales's General Hospital, Tottenham, N.

**At 4.30 p.m.**
**June 23**, Mr. J. H. Evans, **Selected Surgical Cases.**

### MEDICAL GRADUATES' COLLEGE AND POLYCLINIC, Chenies Street, W.C.

**At 5.15 p.m.**
**June 22**, Dr. G. A. Sutherland, **Acute Abdominal Attacks in Children.**
**June 23**, Mr. J. H. Evans, **Cancer of the Face.**
**June 24**, Dr. J. W. Carr, **The Treatment of Acute Pneumonia.**
**June 25**, Dr. W. D. Emery, **Lumbar Puncture.**

### LONDON HOSPITAL MEDICAL COLLEGE, Turner Street, Mile End, E.

**At 3 p.m.**
**June 23**, Mr. Jonathan Hutchinson, F.R.S., **Stigmata Insectorum.**

### ROYAL INSTITUTE OF PUBLIC HEALTH, 37 Russell Square, W.C.

Harben Lectures.

**At 5 p.m.**
**June 24**, Professor G. H. F. Nuttall, **Piroplasmosis.**

### LONDON SCHOOL OF CLINICAL MEDICINE, Seamen's Hospital, Greenwich, S.E.

**At 3.15 p.m.**
**June 22**, Mr. Wm. Turner, **Surgical Conditions of the Gall Bladder.**
**At 3.30 p.m.**
**June 24**, Mr. Cargill, **Optic Nerve Atrophy.**

### MOUNT VERNON HOSPITAL, Fitzroy Square.

**At 5 p.m.**
**June 25**, Dr. F. W. Price, **The Treatment of Early Pulmonary Tuberculosis.**

Royal Commission on the Care and Control of the Feeble-minded (Appointed in 1904).—The report, evidence, etc., in eight volumes, making in all over four thousand pages, besides photographic and other plates, is now being printed by Wyman and Sons, Ltd., and will be issued to Parliament and the public at intervals during the next few weeks. The price of the set will be about two guineas.

The report of the proceedings of the National Conference on Infant Mortality, held at the Caxton Hall, Westminster, in March last, has been published by Messrs. P. S. King and Son.

